# Post-surgical Pyoderma Gangrenosum Following Elective Aesthetic Procedures: A Case Series of 10 Patients and a Multidisciplinary Treatment Approach

**DOI:** 10.1055/s-0045-1814165

**Published:** 2026-04-15

**Authors:** Martina Souilljee Birck, Debora Zmuda Padilha, Vanessa Pletsch Brendler Borges, Mariele Bevilaqua, Renan Rangel Bonamigo

**Affiliations:** 1 Department of Dermatology, Santa Casa Hospital, Rio Grande do Sul, Brazil; 2 Department of Oxygen Therapy, Brazilian Institute of Hyperbaric, Rio Grande do Sul, Brazil

**Keywords:** pyoderma gangrenosum, postoperative, aesthetic surgeries

## Abstract

Post-surgical pyoderma gangrenosum (PPG) is an underdiagnosed variant of a neutrophilic dermatosis that mimics postoperative wound infections, delaying correct diagnosis and treatment. This case series presents 10 female patients who developed PPG following elective aesthetic breast and abdominal surgeries. The average onset of symptoms was 11.4 days postoperatively, with painful ulcerated lesions localized to surgical wounds, often misinterpreted as infection. Diagnosis was primarily clinical and confirmed histologically in 50% of cases. Systemic corticosteroids were the cornerstone of therapy, combined with immunosuppressants in 80% of cases. Hyperbaric oxygen therapy was used in six patients, with excellent clinical response. The average time to clinical resolution was 5.7 months, with no recurrence. Despite successful management, all patients developed unaesthetic scarring. This series reinforces the importance of early suspicion of PPG in atypical postoperative wound evolution and highlights the role of multidisciplinary treatment combining immunosuppression and adjuvant wound care modalities.

## Introduction

Post-surgical pyoderma gangrenosum (PPG) is an uncommon and often misdiagnosed variant of neutrophilic dermatosis that occurs following surgical trauma, typically in previously healthy individuals. Clinically, it presents as rapidly progressive, painful ulcers at surgical sites, often mistaken for wound infections, which may lead to unnecessary antibiotic use or surgical debridement, ultimately worsening the condition. 1 2

Although its pathogenesis remains poorly understood, PPG is considered to result from an abnormal immune response in genetically predisposed individuals. The dysregulation of neutrophilic chemotaxis, coupled with aberrant T cell activation, leads to an overproduction of interleukin-17 (IL-17) and subsequent release of proinflammatory cytokines such as IL-1β, IL-8, and tumor necrosis factor-α (TNF-α). These mediators amplify neutrophilic infiltration and tissue destruction. 3 A phenomenon known as pathergy—the development or worsening of lesions after minor trauma or surgery—is characteristic of pyoderma gangrenosum (PG) and particularly relevant in the postoperative setting. 4 Some patients have associated systemic diseases, especially inflammatory bowel disease. 5 6 The diagnosis is primarily clinical, based on exclusion of infections and other ulcerative dermatoses, and it is supported by compatible histopathological findings in selected cases. 7

Treatment usually involves systemic corticosteroids and/or immunosuppressive agents. Adjunctive therapies such as hyperbaric oxygen therapy (HBOT) may be considered, as it promotes tissue healing by increasing oxygen availability in ischemic or inflamed areas. 8

We report a case series of 10 women diagnosed with PPG after elective aesthetic surgeries, highlighting their clinical characteristics, management, and outcomes.

## Case Series

All patients were female and the mean age at diagnosis was 37.9 ± 7.6 years (range: 25–51 years). Endometriosis ( *n* = 2) was the most frequent comorbidity. Regarding chronic medication use, combined oral contraceptives ( *n* = 2) were the most commonly used ( Table 1 ).

**Table 1:** Post-surgical pyoderma gangrenosum and aesthetic procedures: demographic characteristics of patients

Demographic characteristics	*n* = 10
**Age group (in years)**	
20–29	2
30–39	4
40–49	3
>50	1
**Comorbidities**	
Endometriosis	2
Systemic arterial hypertension	1
Obesity	1
Congenital malformation of the urinary tract	1
Nephrolithiasis	1
Fibromyalgia	1
Depression/anxiety	1
**Continuous medication use**	
Combined oral contraceptive	2
Intrauterine device with progesterone	1
Chlorthalidone	1
Amiloride hydrochloride	1
Omeprazole	1
Pregabalin	1
Sertraline	1
Aripiprazole	1
**History of previous surgery**	
Caesarean section	4
Video cholecystectomy	2
Bariatric surgery	1
Appendectomy	1
Abdominoplasty	1
Hysterectomy	1
**Family autoimmune history**	
Systemic lupus erythematosus (third-degree relative)	1

A history of previous cesarean section was the most frequently reported prior surgery ( *n* = 4), followed by laparoscopic cholecystectomy ( *n* = 2). One patient had a history of abnormal postoperative wound healing after cesarean section, requiring surgical re-intervention. A family history of autoimmune disease was present in only one case (systemic lupus erythematosus in a third-degree relative). None of the patients had a personal history of autoimmune disorders ( Table 1 ).

**Fig. 1:**
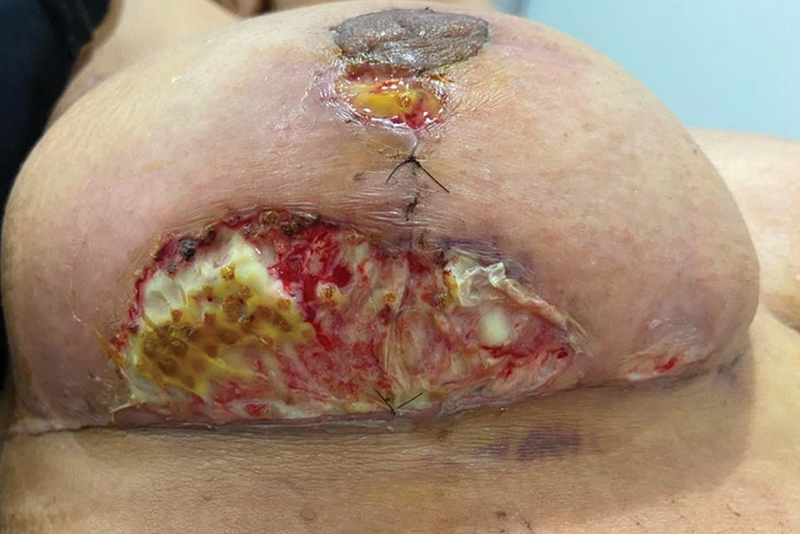
Purulent ulcers along the surgical wound, sparing the nipple, in a 39-year-old patient with PPG, appearing 10 days after reduction mammoplasty. PPG, Post-surgical pyoderma gangrenosum.

Among the surgeries triggering PPG, breast augmentation with implants ( *n* = 4) and reduction mammoplasty ( *n* = 4) were most commonly associated ( Table 2 ). Clinical findings included erythematous papules progressing to plaques and ulcerations ( *n* = 10) along the surgical wound, which were painful ( *n* = 5), purulent-draining ( *n* = 3), or necrotic ( *n* = 1). Lesions appeared on average 11.4 ± 5.7 days postoperatively (range: 5–21 days; Figs. 1 2 3 ). Only one patient presented systemic symptoms, such as fever ( Table 2 ). Bilateral breast involvement occurred in 75% of patients undergoing breast surgery, and one patient experienced implant extrusion ( Fig. 4 ). The nipple–areolar complex was spared in all cases.

**Table 2:** Clinical and diagnostic features of post-surgical pyoderma gangrenosum

Clinical and diagnostic features	*n* = 10
**Type of surgery performed**	
Breast reduction	4
Breast augmentation with implants	4
Liposuction	1
Mastopexy without implants	1
**Symptoms onset (in days)**	
<7	1
7–15	7
16–21	2
**Clinical manifestations**	
Cutaneous manifestations only	9
Complaint of pain	5
Skin lesions with systemic symptoms	1
**Laboratory findings (onset of symptoms)**	
Leukocytosis without juvenile forms	4
Leukocytosis with juvenile forms	1
Anemia	1
Elevated inflammatory tests (ESR/CRP)	3
Reactive RF	2
**Elapsed time to diagnosis (in days)**	
<10	2
<30	3
30–60	4
>60	1
**Histopathology performance for diagnostic aid**	
Yes	5
No	5

Abbreviations: ESR/CRP, erythrocyte sedimentation rate/C-reactive protein; RF, rheumatoid factor.

**Fig. 2:**
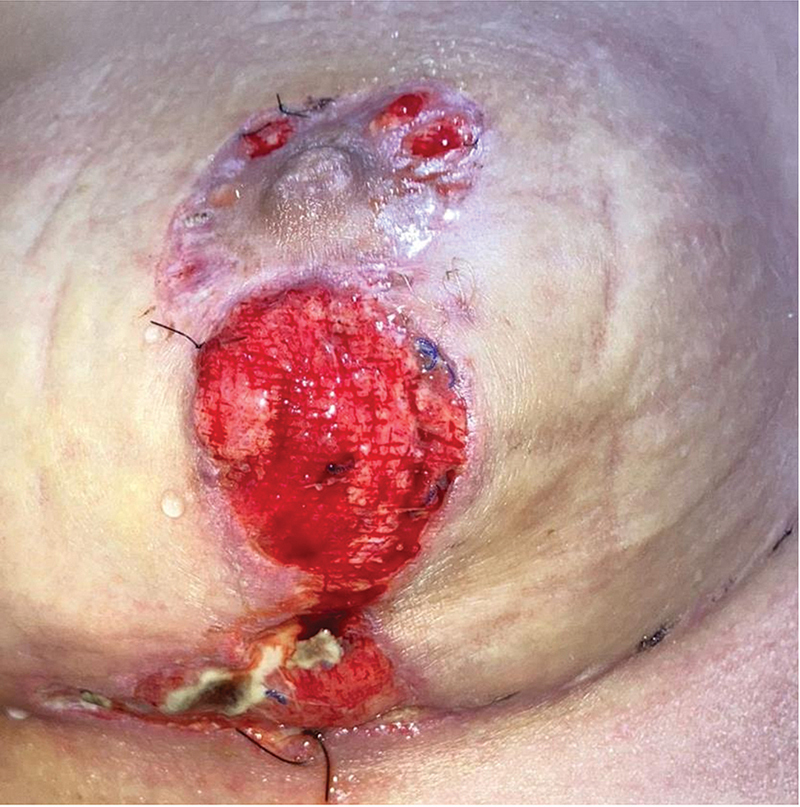
Ulcers along the surgical wound, with violaceous edges, sparing the nipple, in a healthy 25-year-old patient with PPG, 10 days after reduction mammoplasty. PPG, post-surgical pyoderma gangrenosum.

**Fig. 3:**
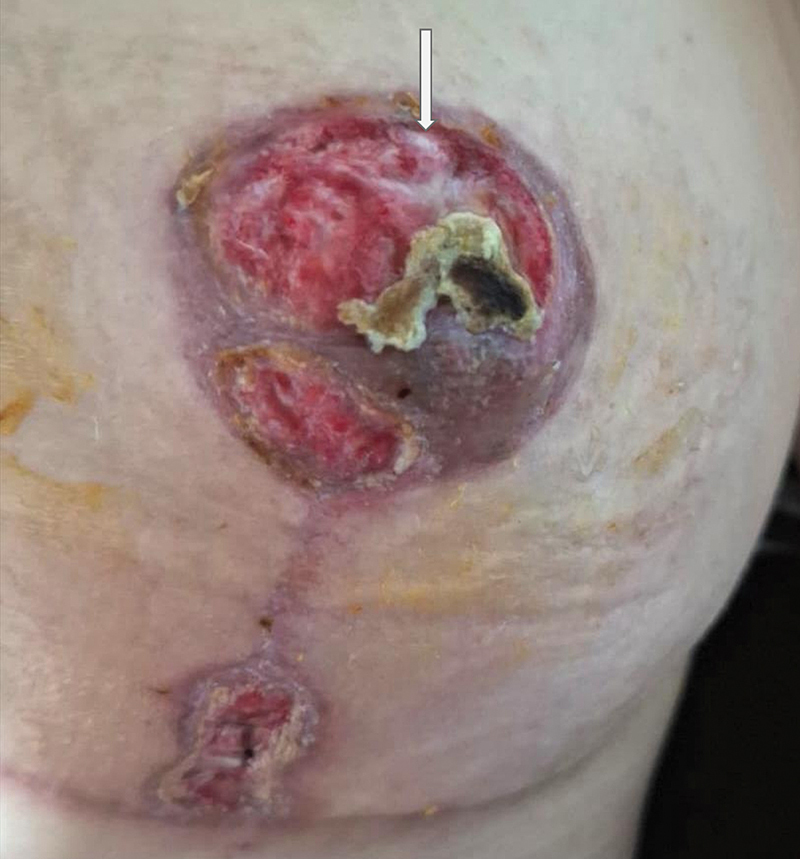
Ulcers along the surgical wound, with violaceous and undermined edges, sparing the nipple, in a 35-year-old patient, 7 days after reduction mammoplasty. The arrow represents Gulliver's sign: epithelial growth at the edge of the ulcer toward the center, leaving cribriform scars.

**Fig. 4:**
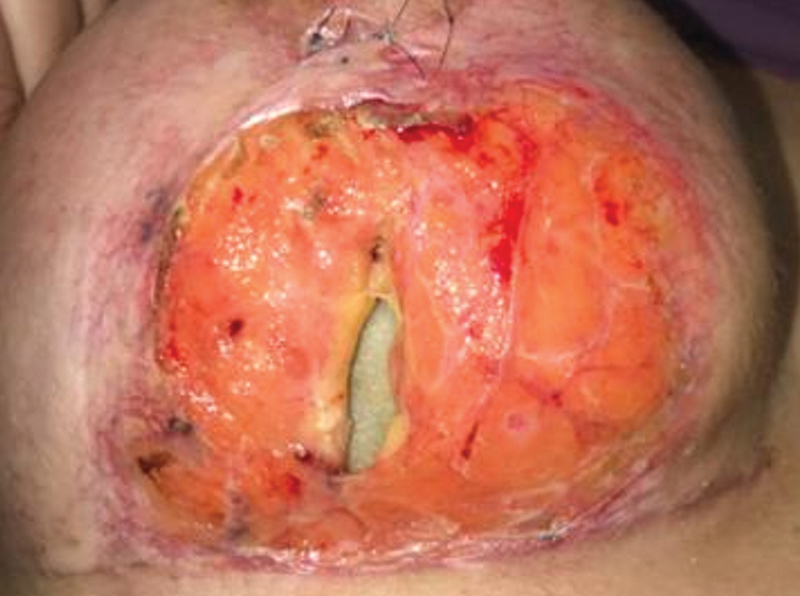
Breast implant extrusion.

All patients underwent laboratory testing after lesion onset, including viral serologies, autoimmune screening (antinuclear antibody, rheumatoid factor [RF], and thyroid function), and wound/secretion cultures. The most common laboratory finding was leukocytosis (WBC > 10,000/µL) without immature forms ( *n* = 4), followed by elevated inflammatory markers ( *n* = 3). RF was reactive in two patients, with values of 10 and 48 IU/mL (nonreactive < 8 IU/mL). Culture results were negative in all cases. Only one patient did not receive antibiotics prior to PPG diagnosis, and half of the patients ( *n* = 5) underwent surgical reintervention, such as resuturing or debridement, which led to worsening of the condition ( Table 2 ).

The diagnosis of PPG was primarily clinical and was established on average 36.7 days (range: 7–150 days) after the surgical procedure. Skin biopsies were performed in 50% of cases ( *n* = 5), mainly to exclude differential diagnoses ( Table 2 ).

Following diagnosis, systemic corticosteroids were the mainstay of treatment (prednisone, *n* = 9), with intravenous methylprednisolone pulses administered in two cases. In 8 of 10 cases, immunosuppressive agents were added to corticosteroids, and two patients required a combination of three medications for disease control. Adjunctive agents included dapsone ( *n* = 4), cyclosporine ( *n* = 4), azathioprine ( *n* = 2), and methotrexate ( *n* = 1). One patient achieved remission with topical therapy alone (clobetasol propionate and tacrolimus). Adjunctive therapies included HBOT, with a mean of 37 sessions (range: 20–60), and low-level laser therapy in one case (6 sessions; Table 3 ). As the procedure was performed at an external facility, detailed technical parameters of the laser device are not available.

**Table 3:** Treatments performed and treatment response

Elected treatment	*n* = 10
Systemic corticosteroid	9
Dapsone	4
Cyclosporine	4
Azathioprine	1
Methotrexate	1
Topical corticosteroid/tacrolimus	1
**Adjuvant therapy**	***n*** ** = 10**
HBOT	6
Low-frequency laser therapy	1
None	3
**Treatment response**	***n*** ** = 10**
Discontinuation of treatment due to remission	9
Still undergoing treatment	1
**Time to remission (in months)**	***n*** ** = 9**
<3	1
3–6	4
>6	4

Abbreviation: HBOT, hyperbaric oxygen therapy.

The mean time to complete wound healing, followed by tapering and discontinuation of therapy, was 5.7 ± 2.6 months (range: 2–10 months). At the time of this publication, one patient remained under treatment with a gradual corticosteroid taper and good clinical response ( Table 3 ).

## Discussion

PPG represents a diagnostic challenge, particularly following elective surgeries. The appearance of rapidly progressing ulcerative lesions with purulent discharge post-surgery is frequently mistaken for common bacterial infection. However, the hallmark violaceous undermined borders, rapid progression despite antibiotics, absence of bacterial growth in cultures, and worsening after surgical debridement should raise suspicion, which requires a markedly different therapeutic approach. 1 2 The pathergy phenomenon is well-documented and can be triggered by minor injuries, including biopsies. 4 9 This risk likely contributed to the low rate of biopsy in our series and underscores the importance of clinical diagnosis and exclusion of infection.

The etiopathogenesis of PG is still not fully elucidated, but growing evidence supports its classification within the spectrum of autoinflammatory neutrophilic syndromes. 10 Genetic predisposition, dysregulated neutrophil function, and the release of inflammatory cytokines—particularly IL-17, IL-1β, TNF-α, and IL-8—promote excessive neutrophilic infiltration and tissue necrosis. 3 Aberrant activation of inflammasomes and the JAK-STAT pathway have been described, providing a rationale for emerging targeted therapies with JAK inhibitors. 11 These mechanisms explain why local trauma, including surgical incisions or debridement, may trigger or exacerbate lesions through the pathergy phenomenon.

Among elective plastic surgeries, breast procedures are the most common triggers for PPG, though the reasons remain unclear. Bilateral breast involvement was observed in 75% of cases, with nipple sparing noted in all patients, a potentially useful diagnostic clue. Fever and leukocytosis were uncommon, which aligns with the understanding that systemic inflammatory signs may be mild or absent in PPG. 12 13

Management requires prompt immunosuppression to control inflammation and prevent tissue destruction. Systemic corticosteroids are considered the first-line treatment, alone or combined with other immunosuppressants like cyclosporine, azathioprine, and dapsone, as applied in our series. 2 6 Our results align with existing literature demonstrating that combination therapy often yields better control of refractory cases. 4 When corticosteroid monotherapy proved insufficient, additional immunosuppressants were selected based on clinical profile, comorbidities, and drug tolerance: cyclosporine was chosen in cases requiring rapid disease control or corticosteroid-sparing effect, given its potent inhibition of T cell activation and cytokine release 14 ; dapsone was used for its anti-neutrophilic and anti-oxidative properties, making it suitable for milder or localized lesions and as an adjunct to corticosteroids 15 ; azathioprine served as a maintenance agent in chronic or relapsing cases, particularly when tapering corticosteroids 3 ; and methotrexate was reserved for refractory disease and in patients intolerant to cyclosporine or dapsone. 10

**Fig. 5:**
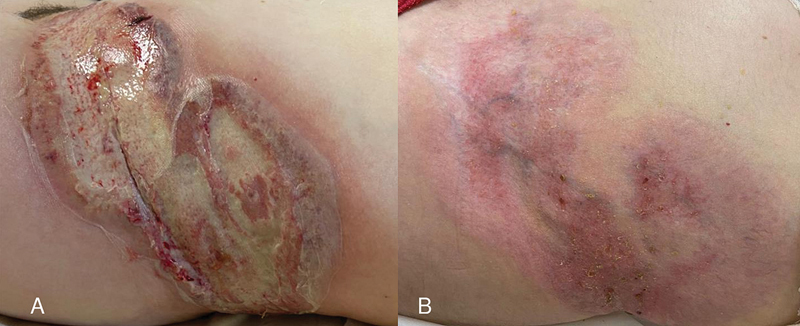
A 40-year-old patient, before ( **A** ) and after ( **B** ) 48 HBOT sessions associated with pulse therapy with methylprednisolone (500 mg/day for 3 days), followed by prednisone (2 mg/kg/day), cyclosporine (200 mg/day), and azathioprine (100 mg/day), gradually reduced over 6 months. Extensive, but not deep, ulceration along the flank liposuction wound. HBOT, hyperbaric oxygen therapy.

Adjunct therapies such as HBOT have shown promise in enhancing wound healing by improving tissue oxygenation, modulating immune responses, and promoting angiogenesis, without provoking pathergy, making it a safe and effective complement to systemic immunosuppression. 6 8 HBOT, administered to several patients in our study, was associated with lesion resolution and good tolerance, echoing findings from earlier case series and reviews ( Fig. 5 ). 7 12

Newer biologic and small-molecule agents, including anti-TNF, anti-IL-1, and JAK-STAT inhibitors, have shown promise in refractory cases, particularly in patients with underlying systemic inflammatory diseases. 11 However, randomized controlled trials remain lacking, and therapeutic choices currently rely on clinician expertise and individual response.

## Conclusion

This case series reinforces that PPG is a diagnostic and therapeutic challenge requiring clinical vigilance and multidisciplinary coordinated care. Prompt initiation of immunosuppressive therapy, careful surgical planning, and adjunctive modalities such as HBOT contribute to improved healing and reduced recurrence. Further studies are warranted to refine treatment protocols and better understand the immunopathogenesis of this serious complication.
